# Successful treatment of canine infective endocarditis caused by *Bacillus amyloliquefaciens*

**DOI:** 10.1080/01652176.2022.2033879

**Published:** 2022-02-11

**Authors:** Hyeona Bae, Tae-Sung Hwang, Hee-Chun Lee, Dong-In Jung, Sang-Hyun Kim, DoHyeon Yu

**Affiliations:** College of Veterinary Medicine, Gyeongsang National University, Jinju, Republic of Korea

**Keywords:** Dog, canine, endocarditis, *Bacillus amyloliquefaciens*, treatment

## Abstract

Bacillus amyloliquefaciens is a gram-positive bacterial species that is utilised as a probiotic in humans and animals. There are no reports of infective endocarditis (IE) in dogs. An 8-year-old, spayed, female Maltese presented with a 1-month history of fever, depression, weight loss, and hindlimb lameness. Laboratory test results indicated non-regenerative anaemia, neutrophilia, hyperglobulinemia, and proteinuria. Echocardiography revealed vegetation on the septal leaflet of the mitral valve and thromboemboli in the left atrium. Consecutive blood culture results revealed that the blood samples were consistently positive for Bacillus amyloliquefaciens, which is generally considered a probiotic bacterial species for animals. Broad-spectrum antibiotics (amoxicillin-clavulanic acid and cefotaxime) and anticoagulants (clopidogrel and rivaroxaban) were administered for 4 months. The clinical signs were responsive to antibiotic treatment. After 4 months, the dog was no longer febrile and the size of the thromboemboli in the left atrium had decreased. Bacteria were no longer isolated in blood cultures after antibiotic therapy. To the best of our knowledge, this is the first case report of canine IE caused by bactaeremic infection with Bacillus amyloliquefaciens.

An 8-year-old, spayed, female Maltese dog presented with a 1-month history of fever, depression, weight loss, and hindlimb lameness. The patient had no particular history of illness. Despite symptomatic treatment at a local veterinary clinic, the fever persisted. The dog was living indoors except for daily walks outside. Dental scaling 2 years previously was the only known medical history.

On physical examination, the dog was febrile (39.3 °C) and had generalised lymphadenopathy. On cardiac auscultation, left-side gallop sounds were detected without murmurs. No dental tartar was found. Infective endocarditis (IE) had to be ruled out initially; therefore, blood culture, echocardiography, and laboratory analyses were performed.

Increased C-reactive protein (66 mg/L; reference range, 0–10 mg/L), mild neutrophilia (12.2 G/L; reference range, 3.0–11.6 G/L) with lymphopenia (0.74 G/L; reference range, 1.1–5.1 G/L), and moderate normocytic normochromic anaemia indicated chronic systemic inflammation. Other possible causes of systemic inflammation were excluded using the SNAP cPL kit (Canine SNAP cPL; IDEXX Laboratories Inc., Westbrook, ME, USA) for pancreatitis and SNAP 4Dx kit (SNAP 4Dx Plus; IDEXX Laboratories Inc.) for canine ehrlichiosis, anaplasmosis, lyme borreliosis, and dirofilariasis. An additional tick/vector polymerase chain reaction test (Canine Tick/Vector Comprehensive RealPCR Panel; IDEXX Laboratories Inc.) was performed with negative results. Serum biochemistry showed mildly increased alkaline phosphatase activity (279 U/L; reference range, 23–212 U/L) and hyperglobulinemia (49 g/L; reference range, 23–45 g/L). A complete urinalysis demonstrated an increased urine protein to creatinine ratio (1.53; reference range, 0.2–0.5), suggesting proteinuria (Figure 1). Additional urine culture results were negative. Since systemic inflammation can affect coagulation, plasma fibrinogen and coagulation screening using thromboelastography (TEG 5000 Hemostasis Analyzer; Haemonetics Corp, Braintree, MA, USA) were performed. The results indicated that the plasma fibrinogen concentration was increased (5470 mg/L; reference range, 2000–4000 mg/L). Furthermore, thromboelastography revealed reduced K time (clotting time), increased alpha angle (the rate of initial clotting to reach an amplitude of at least 20 mm), and increased maximum amplitude (MA) (the maximal width or strength of the clot is increased), indicating a hypercoagulable state (Willard and Tvedten 2011; Villiers and Ristić 2016). Because one of the possible causes of fever of unknown origin and lymphadenopathy is lymphoma (Burgueño and Abreu 2020), the flow cytometric assay for immunophenotyping with CD3-FITC (PC3/188A clone; Santa Cruz Biotechnology, Santa Cruz, CA, USA) and CD21-RPE (CA2.1D6 clone; Bio-Rad Laboratories, Hercules, CA, USA) was used to analyse samples obtained using fine-needle aspiration of superficial lymph nodes, indicating a heterogeneous population of lymphocytes (data not shown) that could eliminate lymphoma from the differential diagnosis list.

**Figure 1.**
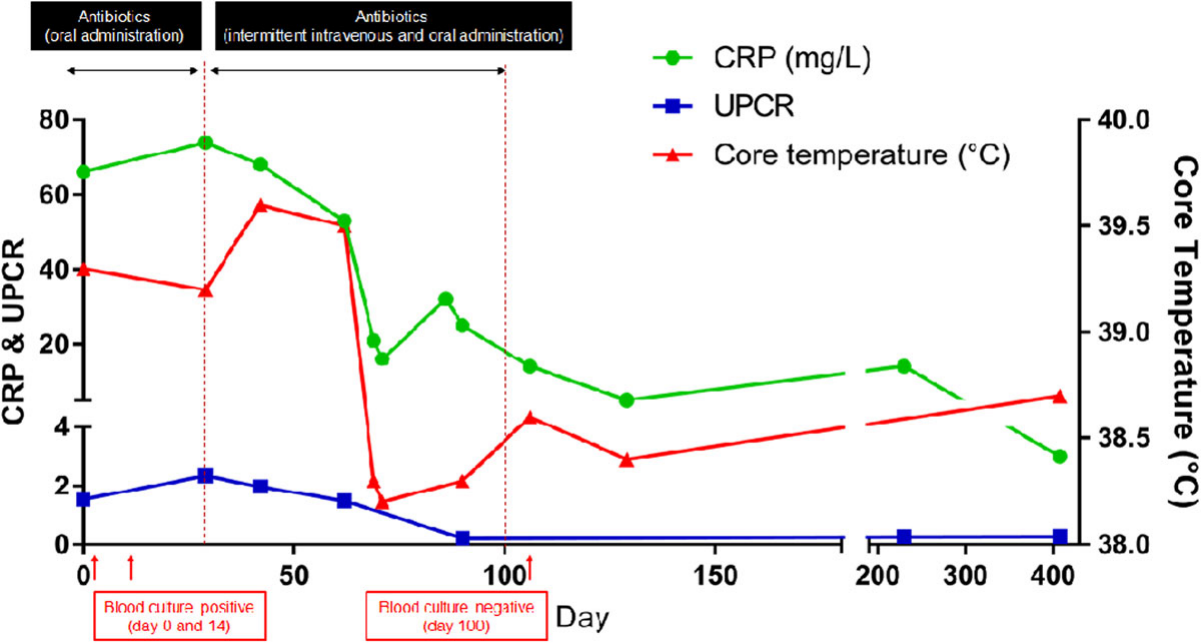
Changes in C-reactive protein (CRP), ratio of urine protein to creatinine (UPC), and body temperature during the therapeutic monitoring period after the diagnosis of infective endocarditis (IE) in a 8-year-old, spayed, female Maltese dog. After the diagnosis of IE, oral administration of the antibiotics showed no improvement in CRP (reference range, 0.1–1 mg/L), UPC (reference range, 2–5 mg/L), body temperature, and clinical signs. Intravenous administration of the antibiotics resulted in blood culture negative, and there were improvements in body temperature, UPC, and CRP levels.

For left-side cardiac gallop sounds, electrocardiography, thoracic radiography, and echocardiography examinations were performed. Electrocardiogram (performed for 30 s) and thoracic radiography findings were unremarkable. However, two-dimensional echocardiography showed a thickened anterior leaflet of the mitral valves and irregular and hyperechoic vegetative lesions on the noncoronary cusp of the aortic valves (Figure 2(A,B)). The left atrium had mild dilation (ratio of the left atrial dimension to the aortic annulus dimension, 1.65), and an echogenic substance that was assumed to be an intra-atrial thrombus was revealed (Figure 2(A,C)). Colour flow Doppler showed regurgitated flow to the left atrium during systole. There was no sign of aortic valvular stenosis or insufficiency, and the right heart was normal, without tricuspid valve lesions. Based on these findings, a tentative diagnosis of aortic and mitral valve endocarditis was made. However, differential diagnosis of mitral regurgitation—myxomatous mitral valve disease (MMVD)—could not be completely excluded.

**Figure 2.**
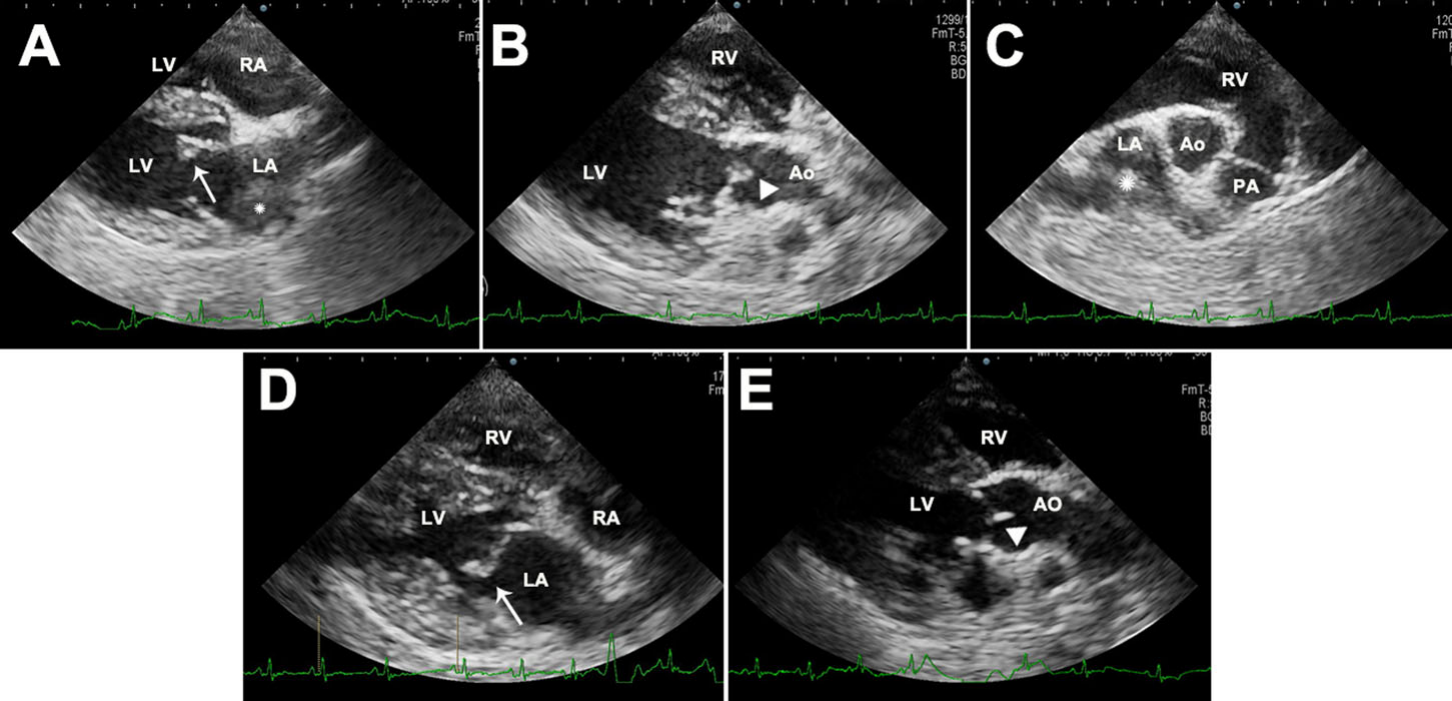
Two-dimensional echocardiographic images of the right parasternal long-axis four-chamber view (A), right parasternal long-axis five-chamber view (B), right parasternal short-axis view (C), thickened anterior leaflet of mitral valves (arrow), and the noncoronary cusp of aortic valves (arrow head) with irregular and hyperechoic vegetative lesions in a 8-year-old, spayed, female Maltese dog. Left atrial thrombus was confirmed (asterisk). Echocardiogram images of the right parasternal long-axis four-chamber view (D) and right parasternal long-axis five-chamber view (E) after 4 months follow-up. The thickening of anterior leaflet of mitral valves (arrow) was still identified, but vegetative lesions of the noncoronary cusp of aortic valve (arrow head) were not found. Decreased size of thrombus was confirmed in the left atrium. LV, left ventricle; RV, right ventricle, LA, left atrium, RA, right atrium; Ao, aorta.

To confirm IE, blood samples were obtained from three different sites based on the Duke criteria (Macdonald 2014). Aseptic venepuncture was undertaken three times to collect 3 mL of blood over a minimum of 1-hour intervals. The blood culture samples, enriched for overnight incubation at 37 °C were streaked onto blood agar plates to isolate bacterial colonies (Figure 3(A)). Each blood culture, whether kept under aerobic and anaerobic incubation conditions, resulted in morphologically identical bacterial colonies (Figure 3(C,D)). Bacterial identification of the isolates was performed using bacteriological procedures, including Gram staining, 16S rDNA sequencing, and spore staining. The identification scheme was added to the basic ID data obtained from automated bacterial ID systems (VITEK®2 system, MALDI Biotyper; Bruker, Billerica, MA, USA). The isolates were identified as *B. amyloliquefaciens*.

**Figure 3.**
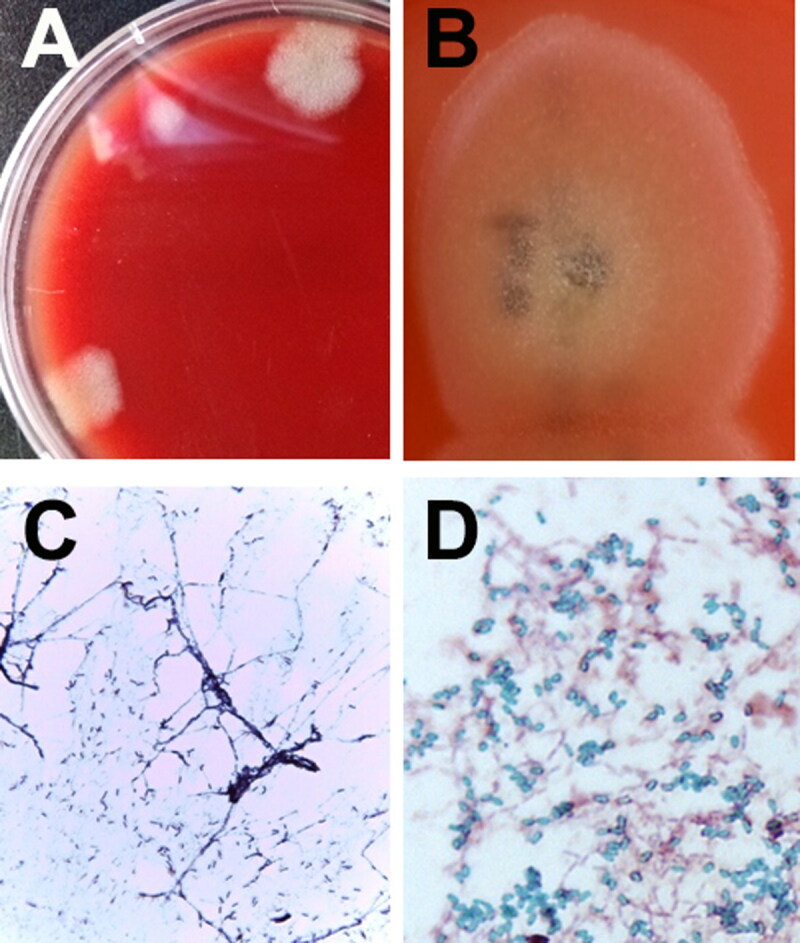
Identification of bacterial isolates from an 8-year-old, spayed, female Maltese dog with infective endocarditis. Initial bacterial colony morphology (A) on the blood agar plate after enrichment in blood culture bottles under aerobic and anaerobic incubation conditions. Close-up appearance of characteristic *Bacillus amyloliquefaciens* colonies (B) grown on blood agar plates (see white, fluffy, wool-like appearance of the colony). Gram-staining (C) and spore-staining (D) of the bacteria grown on the nutrient agar surface (C) and in nutrient broth (D). The results of the bacterial strain showed that the bacteria were somewhat entangled, forming filamentous branches (C). Numerous malachite green-stained *Bacillus* spores were formed in the broth (D).

Based on the medical history, physical examination results (fever, depression, and gallop sounds on auscultation), laboratory test results (high C-reactive protein, leucocytosis, hyperglobulinemia, increased fibrinogen concentration, proteinuria, and hypercoagulable state according to thromboelastography), and echocardiography results (vegetation on the mitral valve and aortic valve), the dog was diagnosed with systemic inflammation caused by IE, and a microbiological analysis confirmed bacteraemia.

The therapeutic strategy focused on antimicrobial and antithrombotic therapy. According to the results of the antibiotic sensitivity tests, the dog was treated with amoxicillin-clavulanic acid (Clavamox; Zoetis Inc., Parsippany, NJ, USA) 12.5 mg/kg BW orally (PO) every 12 hours (q12h) and cefaclor (Cefaclor Cap.; Chongkundang, Seoul, Korea) 15 mg/kg BW PO every 8 hours (q8h) for the first 14 days. An anti-platelet agent (clopidogrel 1 mg/kg BW PO every 24 hours) and direct anti-factor Xa agent (rivaroxaban 1.5 mg/kg BW PO q12h) were administered immediately for prophylaxis of the disseminated intravascular coagulation. Because of the high risk of sudden death due to left atrial thrombi, recombinant tissue plasminogen activator (1 mg/kg BW) was intravenously [IV] administered every 1 h for a total of 10 doses. Pimobendan (0.25 mg/kg BW PO q12h) was administered as inodilator, and benazepril (0.25 mg/kg BW PO every 24 h) was administered for preventing proteinuria. Clinical signs such as fever and anorexia worsened, despite providing a 14-day antimicrobial therapy, and *B. amyloliquefaciens* was re-isolated from the blood culture on day 14. Changing the antibiotics to clindamycin (30 mg/kg BW PO q12h) and enrofloxacin (20 mg/kg BW PO every 24 hours) for an additional 30 days did not improve the clinical signs. Thereafter, the antibiotic route was changed from oral to off-label IV use (amoxicillin-clavulanic acid, 20 mg/kg BW IV q8h; and cefotaxime, 45 mg/kg BW IV q8h) intermittently (31 days in total), and the same drugs were prescribed for oral use for the remaining period; thereafter, the fever resolved and body temperature returned to the normal range. Clinical signs completely resolved after 120 days. Serum globulin and C-reactive protein concentrations also returned to the reference range after 100 days of therapy, and proteinuria was no longer identified (Figure 1). The blood culture results conducted on day 100 were negative, which was at 8 weeks after changing the route of antibiotic administration. After continuous administration of clopidogrel and rivaroxaban for 120 days, echocardiography showed decreased thrombi in the left atrium (Figure 2(D)). As the thickening of the anterior leaflet of the mitral valves was still identified, concurrent MMVD was highly suspected. Nevertheless, vegetative lesions of the noncoronary cusp of the aortic valve were absent (Figure 2(D,E)). To date, the dog has been healthy and non-febrile during follow-up. The hindlimb lameness gradually worsened despite the improvement of other clinical signs according to drug administration. Especially, the hindlimb lameness was confirmed as a degenerative joint disease after continuous follow-up examination.

This case report describes IE in a dog that was successfully treated with antibiotics and antithrombotic agents. *B. amyloliquefaciens* was the causative bacterial species. To the best of our knowledge, this is the first case report of IE in a dog.

However, the exact route of infection could not be confirmed. Among the common causes of bacteraemia are discospondylitis, prostatitis, pneumonia, urinary tract infection, pyoderma, periodontal disease, and long-term indwelling central venous catheters (Macdonald 2014). In this dog, the diseases mentioned were initially ruled out, and there was no recent history of catheterisation. In addition, issues related to poor oral hygiene were less likely to occur because of regular dental management by the local veterinary clinic and tooth brushing. Nevertheless, the source of infection was speculated based on the clinical signs, the cardiac abnormalities, and the bacterial species isolated from a blood culture. Gram-positive bacteria other than *Bacillus* species were found to be the common causative agents of IE (Vogkou et al. 2016). Although rare cases of probiotic *Bacillus*-associated IE were reported (Elshaghabee et al. 2017), more attention should be paid for the probiotic use of the *Bacillus* species because of its spore-forming property and its increasing utilisation as animal feed additives (Hong et al. 2005). The exact translocation mechanism of the bacteria from the gut to the bloodstream remains to be determined. However, it is speculated that the spores may have a role in the translocation process (Gopal et al. 2015). At the time of diagnosis, the dog had a recent history of diet change and had consumed various types of treats for which the exact ingredients were not known. Although there is no evidence that the isolated bacteria came from the probiotics in this case, careful attention would be needed in cases where spore-forming *Bacillus* spp. are administered as probiotics to hospitalized or immunocompromised patients.

IE is uncommon in dogs, but it has a high mortality rate and a short survival time (Sykes et al. 2006). IE is caused by bacterial colonisation within the endocardium and involves the valve leaflets, chordae tendinae, or mural surfaces. The diagnostic criteria for IE are generally based on clinical, microbiological, and echocardiographic observations. Long-term treatment with bactericidal antibiotics has been proposed as a basic intervention for IE. The causative microorganisms, *Staphylococcus* spp., *Streptococcus* spp., *Escherichia coli*, and *Enterococci*, are similar to those involved in IE cases in humans (Fabri et al. 2006; Micol et al. 2006; Peddle and Sleeper 2007; Peddle et al. 2009).

Bacteria of the genus *Bacillus* are widely distributed in the environment as soil saprophytes (Hong et al. 2005). Except for some pathogenic species of *Bacillus*, such as *B. anthracis* and *B. cereus*, the ubiquitous spore-forming, Gram-positive rod-shaped bacteria are part of the normal intestinal flora in humans and animals (Sorokulova 2013; Ravine 2019). *Bacillus amyloliquefaciens* is frequently isolated from fermented soybean foods and can be utilised as probiotics for humans and animals (Lee et al. 2017). Although some food-related *Bacillus* species (spp.) such as *B. cereus*, *B. subtilis*, and *B. licheniformis* have been associated with infectious complications, *B. amyloliquefaciens* has not been associated with disease in humans and animals (Elshaghabee et al. 2017). However, there are safety concerns associated with the utilisation of *Bacillus* spp. as probiotics (Elshaghabee et al. 2017; Ravine 2019) because some food additive bacteria on the generally-recognised-as-safe list, such as *Bacillus subtilis* and probiotic *Lactobacillus* spp., have been increasingly recognised as potential opportunistic pathogens, resulting in an increased number of hospitalised and/or immunocompromised patients (Oggioni et al. 1998; Costa et al. 2018; Yelin et al. 2019).

Because the diagnosis of IE is often ambiguous, several investigators have proposed a series of diagnostic criteria including the modified Duke criteria (Miller et al. 2004; Macdonald 2010; Macdonald 2014). The modified Duke criteria include some major and minor factors: the major factors include positive echocardiogram findings, new valvular insufficiency, and positive blood culture results; the minor factors include fever, medium-to-large–sized dogs (>15 kg), subaortic stenosis, thromboembolic disease, and immune-mediated disease (Macdonald 2014). For this dog, all of the major criteria of the modified Duke criteria were fulfilled. Clinical signs and test results regarding IE improved after medication. However, mitral valve thickening and mild mitral regurgitation were still confirmed. Although vegetative mitral lesions can also induce MR (Macdonald 2010, 2014), mild MR was initially thought to be the sequela of IE. However, although clinical signs and test results regarding IE were improved, mitral valve thickening and MR did not improve. Therefore, it was considered that concurrent degenerative valvular diseases, such as MMVD, may have been present. In this dog, no echocardiography had been performed prior to the diagnosis of IE and there were no clinical signs related to MVD. Thus, the exact time of MVD onset could not be inferred. The gallop sound was audible, which can be confirmed in a diastolic dysfunction characterised by a non-compliant, stiff LV chamber, in general (Fuentes et al. 2010). According to a human study, it is known that diastolic dysfunction occurs in intrinsic cardiac disease and in low-grade inflammation caused by a systemic underlying disease, such as metabolic syndrome, hypertension, diabetes mellitus, chronic obstructive pulmonary disease, anaemia, and chronic kidney disease (Mocan et al. 2019). In this dog, there was direct inflammation of the endocardium, and it was likely that myocardial stiffness and endothelial dysfunction was promoted by inflammatory cytokines, such as IL-6 and TNF-α (Mocan et al. 2019). Therefore, it was hypothesised that the gallop sound was identified because of a transient diastolic dysfunction. Murmur was not audible in this case, which was attributed to the lack of auscultation skill, mild degree of regurgitation jet, or the absence of the murmur.

Based on the bacterial culture and antibiotic sensitivity test, amoxicillin and cephalosporin were used in this case. Admittedly, antimicrobial therapy of *B. amyloliquefaciens* with IE is not well established yet. Synergistic antibiotics (Le and Bayer 2003; Thuny et al. 2012) can be used in cases of refractory bacteraemia that do not respond to standard antibiotic therapy. However, clinicians should attempt to avoid administering multiple antibiotics with a similar mechanism of antibacterial action and spectrum of activity, simultaneously. Furthermore, initial IV antibiotic therapy for 1 to 2 weeks is recommended for powerful therapy in general, followed by long-term oral antibiotic therapy for 6 to 8 weeks or longer (Macdonald 2014). In this case, the oral antibiotics used initially for 45 days were not effective. Immediately after 2 weeks, the administration route was changed to IV, and physical examinations, laboratory testing, and microbial testing were performed. A high dose (20 mg/kg BW q8h) of amoxicillin (IV injection), as is anecdotally used in veterinary medicine, was effective for this dog. However, clinicians are always cautious because IV administration of amoxicillin can cause anaphylactic shock (Norgate and Bruniges 2016), which is indicated by sudden hypotension, oedema, erythema, and pruritus of the facial and extremities 2-3 minutes after administration. Mild erythema was observed a few times in this case, but the clinical signs did not persist. These might be possible limitations of this case.

According to a retrospective study of 71 dogs diagnosed with IE, thromboembolism was detected in 44%. However, thromboembolism was found to be a negative prognostic factor, along with thrombocytopenia, high serum creatinine concentrations, and renal complications (Sykes et al. 2006). The risk of death may double for human patients with thromboembolism (Fabri et al. 2006). Depending on where the thromboembolism occurs, different clinical signs may be present. In this case, blood clots were found in the left atrium; therefore, it was determined that there was a risk of sudden death. Recombinant tissue plasminogen activator was administered to reduce the size of the blood clots, while anticoagulant agents, such as clopidogrel and rivaroxaban (Gagnon et al. 2021; Lo et al. 2021), were administered for long-term management (4 months) to prevent further thrombosis. Anticoagulant and antiplatelet therapies are not recommended because of their trend towards increased haemorrhaging or due to the lack of benefits regarding vegetation resolution or the reduction of embolic events in humans with IE treated with aspirin (Macdonald 2014). The inflammation-induced procoagulant changes seemingly have an important role in thromboembolic complications of IE in humans (Buyukasyk et al. 2004). In contrast, antithrombotic therapy can theoretically reduce the vegetation bacterial density to allow better antibiotic penetration (Peddle and Sleeper 2007). Particularly, for canine patients diagnosed with a hypercoagulable state, management should be provided in advance to prevent thromboembolism (Bae et al. 2019). In this case, a hypercoagulable state could be diagnosed using thromboelastography, and disseminated intravascular coagulation could be prevented. Additionally, the long-term administration of clopidogrel and rivaroxaban was effective and the size of blood clots in the left atrium was significantly reduced. Therefore, it is important to use thromboelastography to check the coagulation status in dogs with systemic inflammation. Despite the lack of evidence of the fibrinolytic effects of clopidogrel and rivaroxaban, these agents yielded a good clinical response in this case. A randomised, controlled clinical study is needed in the future.

The survival rate of IE, which has been assessed in several previous studies, is not high, and the mean survival time is short (Sykes et al. 2006; Macdonald 2014). Moreover, the prognosis is very poor when clinical signs are acute with heart failure, thromboembolism, and renal complication (Miller et al. 2004; Sykes et al. 2006; Peddle and Sleeper 2007; Macdonald 2014). Depending on the affected valve, the prognosis is poor to grave. Dogs with aortic valve involvement have a median survival time of 3 days. However, those with mitral valve disease have a median survival time of 476 days (Macdonald 2010). In cases where *Bartonella* was the causative bacterium, dogs were significantly more likely to be afebrile, had predominantly aortic valve involvement, and had a higher prevalence of congestive heart failure than dogs infected with other pathogens (Sykes et al. 2006). Dogs infected with streptococci, however, were more likely to have mitral valve involvement than dogs infected with other organisms (Sykes et al. 2006). The IE prognosis for *Bacillus*-infected human patients is usually good; no fatalities occurred among the 27 reported patients, and only 2 patients required surgery for valve replacement in one study (Brouqui and Raoult 2001). However, because *B. amyloliquefaciens* has never been reported as the cause of IE, the associated morbidity and mortality are unknown. Initially, the prognosis was considered to be very poor since vegetations were found in both the mitral valve and aortic valve on echocardiography and thromboembolism occurred in the left atrium. According to the negative blood culture results obtained during and after therapeutic treatment with amoxicillin-clavulanic acid, the dog that received IV antibiotics and anticoagulants became free of the bacteria.

Microbiological and echocardiographic findings strongly supported our hypothesis that the isolates of *B. amyloliquefaciens* were the causative bacteria in this case of canine IE. Although it is uncommon for *Bacillus* species to cause IE in animals and humans, attention should be focused on these spore-forming bacteria with probiotic potential. This case report not only conveys meaningful diagnostic and therapeutic considerations, but also draws attention to the possible complications associated with infection by potential probiotic bacteria.
